# Telesurgery in Urology is the Hot Topic in this Number of International Brazilian Journal of Urology

**DOI:** 10.1590/S1677-5538.IBJU.2025.03.01

**Published:** 2025-04-04

**Authors:** Luciano A. Favorito

**Affiliations:** 1 Universidade do Estado do Rio de Janeiro Unidade de Pesquisa Urogenital Rio de Janeiro RJ Brasil Unidade de Pesquisa Urogenital - Universidade do Estado do Rio de Janeiro - Uerj, Rio de Janeiro, RJ, Brasil;; 2 Hospital Federal da Lagoa Serviço de Urologia Rio de Janeiro RJ Brasil Serviço de Urologia, Hospital Federal da Lagoa, Rio de Janeiro, RJ, Brasil

The May-June number of Int Braz J Urol presents original contributions with a lot of interesting papers in different fields: Telesurgery, Prostate Cancer, Endourology, Basic Research, Penile Cancer, Bladder cancer, Urethral Cancer, Urachal carcinoma, Neurophysiology of Micturition, Testosterone replacement therapy and MRI in Pediatric Urology. The papers came from many different countries such as Brazil, Italy, China, Belgium, Turkey, Argentina, Canada and USA, and as usual the editor´s comment highlights some of them. The editor in chief would like to highlight the following works:

Dr. Ferreira and collegues from Brazil, presented id e20240494 (1) a nice narrative review about telesurgery in Urology to review the literature aiming at the surgical success rate as a primary objective, and secondly, the most important patient outcomes and the network system and concluded that despite its limitations, there was evidence demonstrating that robotic surgery in the genitourinary system is safe and feasible, however it is a subject that must be well discussed, and further studies must be carried out.

Dr. Suartz and collegues from performed id e20240665 (2) an interesting narrative review about the urachal carcinoma to consolidate current evidence on the diagnosis, epidemiology, and treatment of urachal carcinoma, a rare malignancy with limited data and concluded that this study provides the most comprehensive review of urachal carcinoma to date, providing evidence to guide clinical decisions. It underscores the oncologic benefits of *en-bloc* resection with umbilectomy and specific chemotherapeutic regimens. Emerging alternative thera- pies also show potential, highlighting the need for further research to optimize patient outcomes.

Id e20250047 (3) we can observed an important review performed by Dr. Press and Dr. Kirsch from USA about the Magnetic Resonance Urogram in Pediatric Urology. This paper is the cover of this edition. Magnetic Resonance Urography (MRU) has emerged as a powerful imaging modality in pediatric urology, offering comprehensive anatomical and functional assessment of the urinary tract without exposure to ionizing radiation. The authors concluded that this review highlights the growing significance of MRU in pediatric urinary tract evaluation, emphasizing its potential to improve clinical decision-making and patient outcomes.

Dr. Basheer and collegues from USA and Brazil permorfed id e20259904 (4) a nice review about the management of Adverse Effects in Testosterone Replacement Therapy to provide the most updated knowledge regarding the treatment of adverse effects secondary to testosterone replacement therapy (TRT), such as gynecomastia, cardiovascular and hematologic risks, prostate health risk, and liver dysfunction risks and concluded that monitoring and management of adverse effects are critical to maximize benefit and minimize the risks of TRT. Ongoing research will further elucidate the safety of TRT while advancing evidence-based practices in managing its associated adverse effects. Effective patient education and counseling are also essential to improve compliance and treatment outcomes.

Dr. Mattos and Favorito from Brazil performed id e20259907 (5) an interesting narrative review about the neurophysiology of micturition and concluded that the interplay between the complexity of LUTF, the widespread prevalence of conditions that can disrupt it, and the nonspecific nature of related symptoms frequently complicate urological decision-making. Overlooking associated neurological factors can result in suboptimal outcomes, diminished quality of life, and serious adverse consequences. A systematic approach is crucial to minimizing the risk of misdiagnosis and mismanagement, especially when considering invasive interventions.

Dr. Özbilen and collegues from Turkey performed id e20240500 (6) a nice multicentric study about the External Validation and Comparison of Current Scoring Systems in Encrusted Ureteral Stent (EUS) and concluded that the management of EUSs is often challenging for urologists. Although the current scoring systems for EUS differ somewhat, it is important to use scoring systems to guide the management of these patients.

Dr. Sighnolfi and collegues from Italy performed id e20240556 (7) a nice study about the Radical Cystectomy with Elective Indication to Cutaneous Ureterostomy (CUS): Single-Center Comparative Analysis Between Open and Robotic surgery (RARC) and concluded that RARC appears to be associated with lower morbidity and reduced incidence of complications, elements that make it particularly suitable for frail patients with an elective indication for CUS.

Dr. Villoldo and collegues from several countries of Latina America performed id e20240615 (8) an interesting multicentric study about the BCG. Use in Non-Muscle Invasive Bladder Cancer in Latin America and concluded that this study highlights critical deviations from recommended NMIBC management protocols in Latin America, including delayed BCG initiation and inconsistencies in maintenance therapy. These findings emphasize the need for standardized treatment protocols and improved adherence to international guidelines, which could enhance NMIBC patient outcomes in the region. Collaborative efforts are essential to develop region-specific strategies, improve data collection, and ultimately provide better care for bladder cancer patients in Latin America.

Dr. Fazili and collegues from Belgium and USA performed id e20259905 (9) a nice study about the role Of DSNB in Staging of Primary Urethral Cancer. The authors show the use of dynamic sentinel node biopsy (DSNB) in five patients with primary urethral squamous cell carcinoma (U-SCC) and no evidence of inguinal node disease. In the five DSNBs performed, clinically occult nodal metastasis was discovered in one patient. In this patient DSNB was performed after local recurrence and repeat imaging confirming cN0 status. Only one minor complication with DSNB was observed. Awaiting further investigations in larger series, this study highlights the feasibility of DSNB in primary U-SCC with clinically node negative disease.

The Editor-in-chief expects everyone to enjoy reading.
